# Perigastric and Portal Venous Gas Induced by Vomiting

**DOI:** 10.5334/jbsr.2300

**Published:** 2020-12-07

**Authors:** Louise Pichon, Pierre-Antoine Poncelet

**Affiliations:** 1 Department of Medical Imaging, Cliniques Universitaires Saint Luc, Avenue Hippocrate 10, 1200 Bruxelles, BE

**Keywords:** Vomiting, hepatoportal venous gas, aeroportia, gastric

## Abstract

**Teaching point:** Portal venous gas is often associated with severe abdominal pathologies, but may be also encountered in less dramatic conditions such as vomiting.

## Case report

A 64-year-old man was admitted to our emergency department due to epigastric pain and several episodes of vomiting. He was hemodynamically stable and had normal abdominal examination. Laboratory findings were without any abnormalities.

A thoraco-abdominal Computed Tomography (CT) scan was performed to rule out aortic dissection. It revealed no dissection but gas in the perigastric and portal veins (Figure A and B, arrows and arrowhead). Gastric and intestinal walls showed normal enhancement. There was neither free fluid nor free air in the abdominal cavity. Taking into account to the good patient condition, a conservative treatment was conducted. A repeat unenhanced CT was performed 12 hours after and showed complete resolution of the venous gas (Figure C).

**Figure A, B, C:**
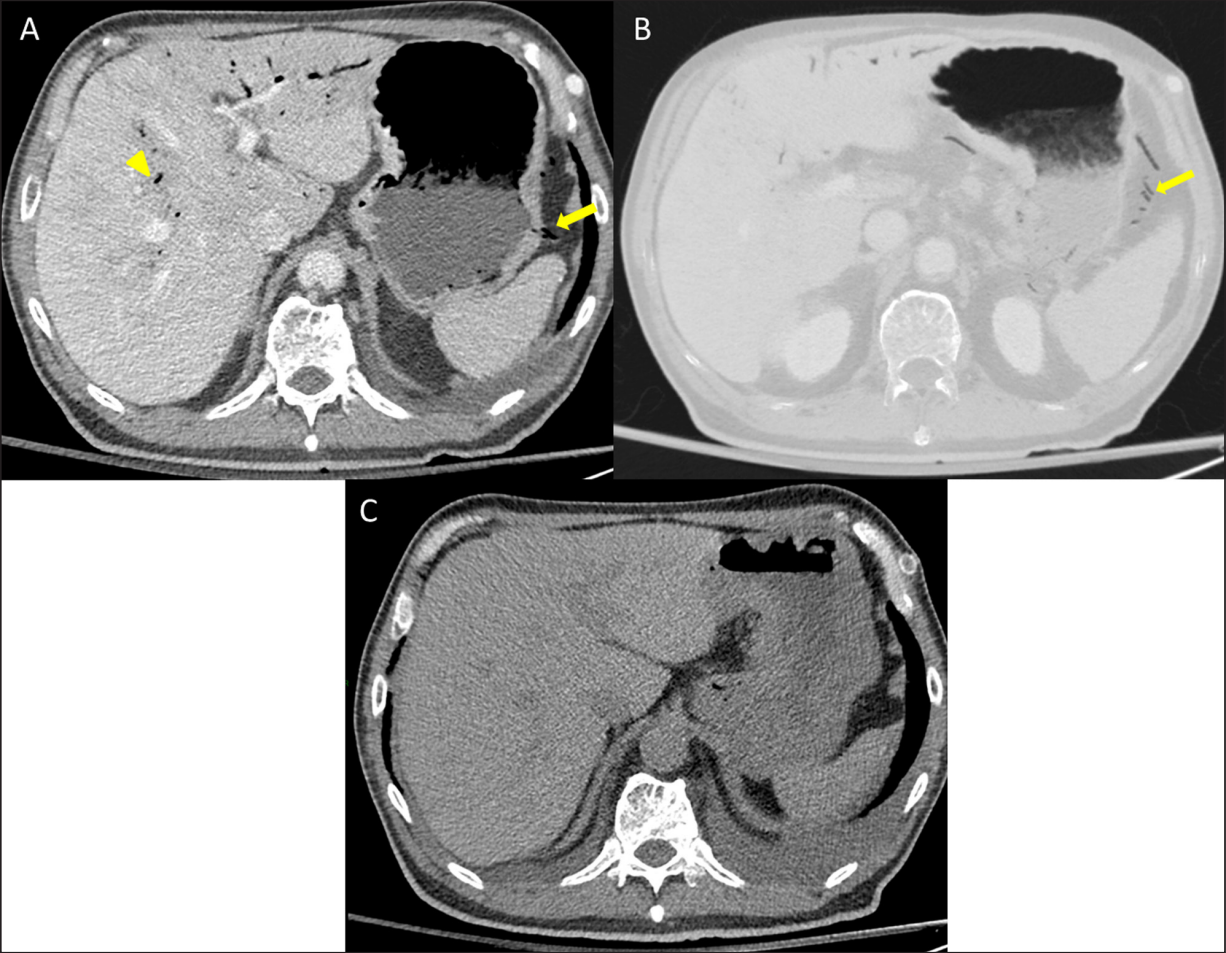
**A)** Axial CT image in portal phase showing portal venous gas (arrowhead) and gas in perigastric veins (arrow). Note the liver parenchyma hypoperfusion around the aeroportia and normal enhancement of gastric wall. **B)** Minip reconstruction showing gas in perigastric veins. **C)** Unenhanced axial CT image performed 12 hours after the vomiting episode showing complete resolutions of venous gas (portal and perigastric).

## Comment

The main theory to explain our CT images is that vomiting induced elevated intra-gastric pressure with translocation of air through the submucosa and spreading to the portal system. The good patient condition and the complete resolution several hours later confirmed the benign etiology. Other causes of perigastric venous air and subsequent aeroportia include mucosal injury (ulcer, trauma, caustic ingestion), infection (emphysematous gastritis) and ischemia [1].

Aeroportia must raise attention for severe abdominal pathology often requiring surgery, such as like bowel ischemia, but it’s important to know that it can be associated with benign lesions, such as the one herein described.
